# Resveratrol Downregulates miR-155-5p to Block the Malignant Behavior of Gastric Cancer Cells

**DOI:** 10.1155/2022/6968641

**Published:** 2022-06-25

**Authors:** Nana Su, Lanlan Li, Erle Zhou, Hong Li, Shuhua Wu, Zhang Cao

**Affiliations:** ^1^Department of Pathology, Binzhou Medical University, Yantai 264003, China; ^2^Department of Pathology, Binzhou Medical University Hospital, Binzhou 256603, China

## Abstract

Studies have shown that resveratrol (Res) exerts significant antiproliferative effects in cancer, and regulating the expression of microRNAs (miRNAs) is one the underlying mechanisms of these effects. Overexpression of miR-155-5p leads to oncogenesis. However, it is unclear whether Res exerts antitumor effects by regulating the expression of miR-155-5p, and its specific mechanism in gastric cancer remains unknown. In this study, qRT-PCR was performed to assess the expression of miR-155-5p in gastric cells and clinical tissues, and the MTT assay, plate clone formation test, cell scratch test, Transwell assay, and flow cytometry were performed to investigate the functions of Res on the growth of gastric cancer cells after treatment with miR-155-5p. Western blot analysis was performed to detect the expression of claudin 1, c-Myc, cyclin D1, Bcl-2, and caspase-3 proteins in gastric cancer cell lines after treatment with miR-155-5p and Res. We found that miR-155-5p was overexpressed in gastric cancer cells and clinical tissues, while Res inhibited gastric cancer cell growth by regulating miR-155-5p expression. The results of MTT assay, plate clone formation test, cell scratch test, Transwell test, and flow cytometry showed that miR-155-5p promoted the proliferation, invasion, and metastasis of gastric cancer cell lines and inhibited apoptosis, while Res addition inhibited this effect (*P* < 0.05). When miR-155-5p was overexpressed, the expressions of claudin 1, c-Myc, cyclin D1, and Bcl-2 were upregulated and that of caspase-3 was downregulated. Collectively, these results suggest that miR-155-5p may be a therapeutic target in gastric cancer, and Res may be a potential therapeutic agent based on its regulation of miR-155-5p.

## 1. Introduction

Gastric cancer was the fifth most frequently diagnosed cancer and the third leading cause of cancer-related death in 2018 [1]. Furthermore, more than 90% of patients with gastric cancer are diagnosed with distant metastases [2]. At present, radiotherapy, chemotherapy, and targeted therapy are the main treatments for gastric cancer. However, chemotherapeutic drugs have high toxicity and low specificity [3, 4]. Therefore, it is imperative to identify effective therapeutic agents.

In recent years, resveratrol (Res), a natural polyphenolic compound widely present in both dietary foods and plant species, has been reported to be an effective natural anticancer drug. Both preclinical investigations and experimental studies have shown that Res can impact tumor initiation and progression in a wide range of malignancies [5]. Therefore, Res may be an ideal anticancer or prophylactic drug [6]. Additionally, some studies have shown that Res can exert its antitumor effect by regulating the expression of microRNAs (miRNAs). For instance, Sheth et al. [7] reported that Res inhibited cell growth and metastasis in prostate cancer by regulating miRNA-21 expression.

miRNAs are endogenous single-stranded noncoding RNAs that bind to the 3′-untranslated regions of target miRNAs and can regulate gene expression at the posttranscriptional level [8]. To date, nearly 3000 miRNAs have been identified in humans, and researchers predict that miRNAs may modulate more than half of all human genes. The roles of miRNAs in cancer biology have been extensively studied over the past decades. Some miRNAs participate in tumor-cell invasion and metastasis [9–12], while others are closely associated with the prognosis of patients with cancer [13–15]. Studies have shown that miR-155-5p is overexpressed in cancers of lungs, breasts, and cervix and is correlated with poor prognosis [14, 16, 17]. However, it is unclear whether miR-155-5p is overexpressed in gastric cancer and whether Res regulates miR-155-5p expression in gastric cancer to block tumor progression. In this study, we examined the role of miR-155-5p and the effect of Res on miR-155-5p in gastric cancer to determine a new therapeutic target for this disease.

## 2. Materials and Methods

### 2.1. Patients and Tissue Samples

Forty-nine tissue specimens were obtained from the Binzhou Medical University Hospital from April 2018 to December 2019. The samples were obtained from 35 men and 14 women. In addition, fresh cancer tissues and paracancerous tissues were obtained from 14 patients with gastric cancer, and tumor tissues were immediately frozen in liquid nitrogen for western blot analysis. This study was approved by the Medical Ethics Committee of the Binzhou Medical University Hospital (Lun Yan Grant No. 2016-26), and all patients provided informed consent. None of the patients had received radiotherapy, chemotherapy, targeted therapy, or other anticancer therapy before surgery.

### 2.2. Cell Culture

The human gastric cancer cell line SGC7901 was donated by the Central Laboratory of the Binzhou Medical University Hospital. GES-1, MGC803, and AGS cell lines were donated by the Laboratory of Pathogen of the Basic Medical College of Binzhou Medical University. MGC803 and GES-1 cells were cultured in high-glucose DMEM supplemented with 10% fetal bovine serum (FBS). AGS and SGC7901 cells were maintained in RPMI 1640 medium supplemented with 10% FBS. Gastric cancer cells were treated with different concentrations (0, 25, 50, 100, and 200 *μ*M) of Res.

### 2.3. Transfection

Oligonucleotides, including scrambled control, hsa-miR-155-5p-mimic, and hsa-miR-155-5p-inhibitor, were synthesized and purified by Takara (Dalian, China). SGC7901 and MGC803 cells were seeded (2 × 10^5^ cells/well) in 6-well plates and transfected with scrambled controls, hsa-miR-155-5p-mimics, or hsa-miR-155-5p-inhibitor using Lipofectamine 2000 (Invitrogen, Carlsbad, CA, USA) according to the manufacturer's protocol. The sequences of these molecules were as follows: miR-155-5p-mimic, 5′-UUAAUGCUAAUCGUGAUAGGGGU-3′; miR-155-5p-inhibitor, 5′-ACCCCUAUCACGAUUAGCAUUAA-3′; and scrambled control, 5′-CCCUAUCACAAUUAGCAUUAAUU-3′.

### 2.4. MTT Assay

The cells showing good growth after transfection were seeded into 96-well plates (3000 cells/well). MTT reagent was added to the culture medium at a final concentration of 0.1 mg/mL. Next, 150 *μ*L dimethyl sulfoxide was added to the cells to dissolve the formazan crystals. Optical density was measured at 490 nm using a SpectraMax M2 microplate reader (Molecular Devices, Shanghai, China).

### 2.5. Colony Formation Assay

After transfection, the cells were seeded in 100 mm Petri dishes (2000 cells/dish). The medium was replenished every 3 days, and the cells were allowed to grow for 12 days to form colonies. The colonies were fixed with 4% paraformaldehyde (Biosharp, Anhui, China), stained with 0.1% crystal violet solution (Solarbio, Beijing, China), and those with >50 cells were counted.

### 2.6. Apoptosis Assay

Apoptosis was analyzed using the FITC-Annexin V Apoptosis Detection Kit I (Becton Dickinson, Franklin Lakes, NJ, USA) according to the manufacturer's protocol. The transfected SGC7901 and MGC803 cells were incubated with Res for 24 h. Next, the cells were collected and washed with phosphate-buffered saline (PBS) precooled at 4°C. The cells (1 × 10^5^) were suspended in 1× Annexin V Binding Buffer, and 5 *μ*L Annexin V-FITC and 5 *μ*L propidium iodide were added to the cell suspension. The samples were incubated at room temperature for 20 min in the dark and then analyzed using a FACSCanto flow cytometer.

### 2.7. Wound Healing Assay

The cells were grown in a 6-well plate until they reached 90% confluence. Scratches were made in the cell monolayer using a 10 *μ*L sterile pipette tip. Next, the cells were washed with PBS, and the medium was replaced with fresh medium supplemented with 1% FBS to inhibit cell proliferation. The scratched areas were photographed at the indicated time points using an Olympus TL4 photomicroscope (Olympus, Tokyo, Japan) and analyzed using ImageJ software (National Institutes of Health, Bethesda, MD, USA).

### 2.8. Transwell Assay

Cell migration and invasion were measured in Transwell membrane chambers with 6.5 mm inserts and 8 *μ*m pore polycarbonate membranes (Corning Inc., Corning, NY, USA). For the invasion assay, the Transwell membrane chamber was layered with Matrigel (Corning) and incubated for 4 h at 37°C. The excess Matrigel was then gently removed. Two hundred-microliter serum-free medium and 600 *μ*L medium containing 20% FBS were added to the upper and lower chambers, respectively. SGC7901 and MGC803 cells (1 × 10^5^ cells/mL) were seeded into the upper chamber and incubated at 37°C for 24 h. Subsequently, the upper chamber was removed, and the cells were wiped on a chamber filter with a cotton swab. After washing with PBS, the membrane was fixed with 4% paraformaldehyde for 1 h and stained with 0.1% crystal violet solution for 15 min. Excess dye was washed away, and the cells were photographed in 5–10 randomly selected fields under a microscope at 400× magnification (Canon, Beijing, China). The cell migration assay was performed as described above, except that Matrigel coating was not added.

### 2.9. Quantitative Real-Time Polymerase Chain Reaction

Total RNA was isolated from cell lines and paraffin-embedded tissue specimens using RNAiso Plus (Takara, Dalian, China) according to the manufacturer's instructions. RNA was stored at -80°C until further use. cDNA was synthesized using the Mir-X miRNA First-Strand Synthesis Kit (Takara). The expression of target genes was detected by performing quantitative real-time polymerase chain reaction (qRT-PCR) using TB Green Premix Ex Taq II (Takara) according to the manufacturer's instructions. The gene expression was subjected to relative quantification using the comparative threshold cycle (Ct) method. The expression of miR-155-5p was analyzed using U6 as an internal control.

### 2.10. Western Blot Assay

Total protein from cells and gastric cancer tissues was extracted using radioimmunoprecipitation assay lysis buffer containing protease inhibitors. Protein samples (20 *μ*g) were resolved by sodium dodecyl sulphate-polyacrylamide gel electrophoresis (12% resolving gel) and then transferred to a polyvinylidene difluoride membrane (Millipore, Bedford, MA, USA). Membranes were then blocked with 5% nonfat milk to block nonspecific binding for 3 h at room temperature and incubated with primary antibodies against claudin 1 (1 : 1000, Abcam, Cambridge, MA, USA), caspase-3 (1 : 1000, Abcam), Bcl-2 (1 : 1000, Proteintech, Rosemont, IL, USA), c-Myc (1 : 1000, Proteintech), cyclin D1 (1 : 1000, Beyotime, Shanghai, China), and GAPDH (1 : 1000, Goodhere, Hangzhou, China) at 4°C overnight, followed by incubation with horseradish peroxidase-conjugated secondary antibody (1 : 2000, ZSGB-BIO, Beijing, China) at room temperature for 3 h. GAPDH was used as an internal control for the relative protein expression. Protein bands were quantified using ImageJ software (NIH).

### 2.11. Statistical Analysis

Statistical evaluation was performed using mean ± standard deviation. Comparisons between two groups were performed using Student's *t*-test, and multiple comparisons were assessed by performing one-way analysis of variance. Among-group differences were examined using a one-way analysis of variance. Statistical significance was set at *P* < 0.05. All experiments were performed in triplicate.

## 3. Results and Discussion

### 3.1. miR-155-5p Is Overexpressed in Gastric Cancer Tissues and Cells, and Res Downregulates miR-155-5p Expression

**Figure 1:**
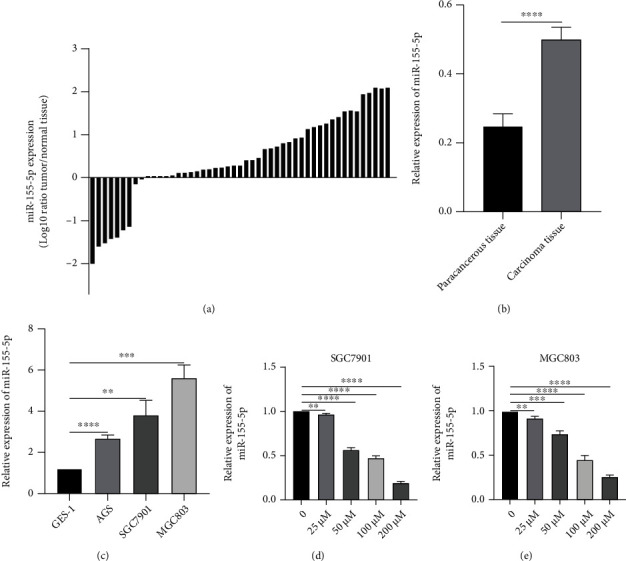
The expression of miR-155-5p in gastric cancer tissues and cells. (a and b) The expression of miR-155-5p in gastric cancer and para-cancerous tissues. (c) The expression of miR-155-5p in gastric cancer cell lines. (d and e) The expression of miR-155-5p in SGC7901 and MGC803 gastric cancer cells treated with different concentrations of Res. ^∗∗^*P* < 0.01, ^∗∗∗^*P* < 0.001, and ^∗∗∗∗^*P* < 0.0001.

Results of qRT-PCR revealed that among the 49 gastric cancer cases, 40 (81.63%) showed high expression of miR-155-5p, whereas 9 (18.37%) had low expression of miR-155-5p (Figure 1(a)). Compared with adjacent normal tissue and gastric mucosal cells, miR-155-5p expression was increased in gastric cancer tissues and gastric cancer cell lines (Figures 1(b) and 1(c)). The expression of miR-155-5p decreased with Res treatment in a dose-dependent manner (Figures 1(d) and 1(e)).

### 3.2. Res Inhibits Proliferation and Promotes Apoptosis in Gastric Cancer Cells

**Figure 2:**
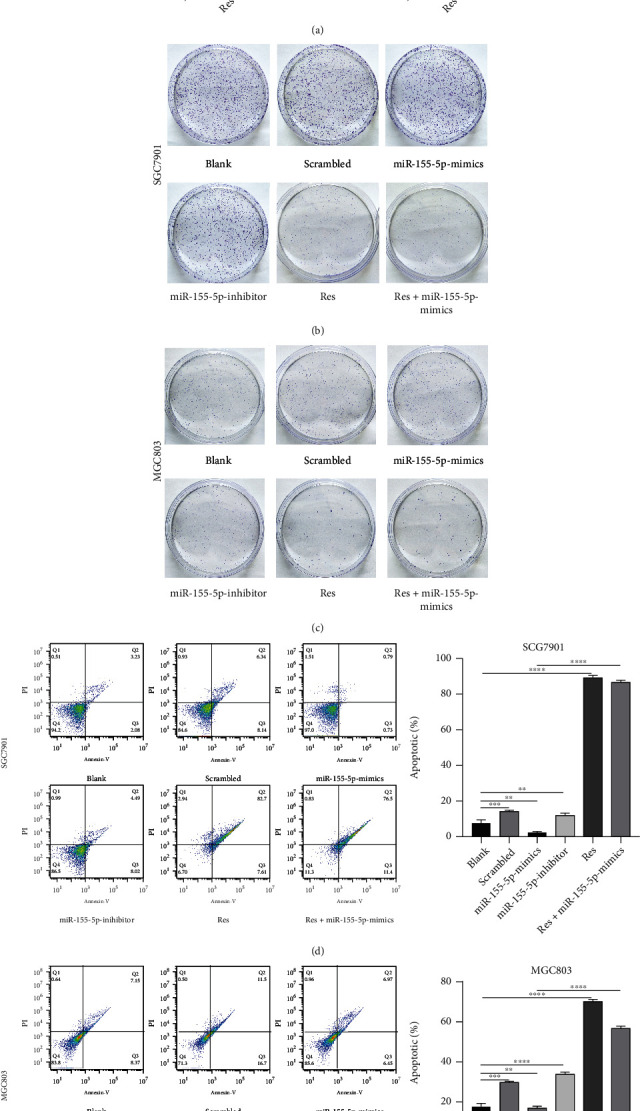
Res inhibits proliferation and promotes apoptosis in gastric cancer cells. (a) Cell viability of SGC7901 and MGC803 cells treated with Res was estimated with MTT assays. (b and c) Colony formation in SGC7901 and MGC803 cells treated with miR-155-5p and 80 *μ*M Res was estimated by MTT assays. (d and e) Apoptosis of SGC7901 and MGC803 cells treated with miR-155-5p-mimics and 80 *μ*M resveratrol. ^∗^*P* < 0.05, ^∗∗^*P* < 0.01, ^∗∗∗^*P* < 0.001, and ^∗∗∗∗^*P* < 0.0001.

MTT and colony formation assays showed that transfection of miR-155-5p-mimics promoted cell proliferation, whereas Res significantly inhibited cell proliferation. Proliferation was also inhibited in cells transfected with miR-155-5p-mimics and treated with Res (Figures 2(a)–2(c)). Apoptosis assays showed that miR-155-5p-mimics inhibited apoptosis, whereas Res treatment significantly increased apoptosis, even in cells transfected with miR-155-5p-mimics (Figures 2(d) and 2(e)).

### 3.3. Res Inhibits Migration and Invasion in Gastric Cancer Cells

**Figure 3:**
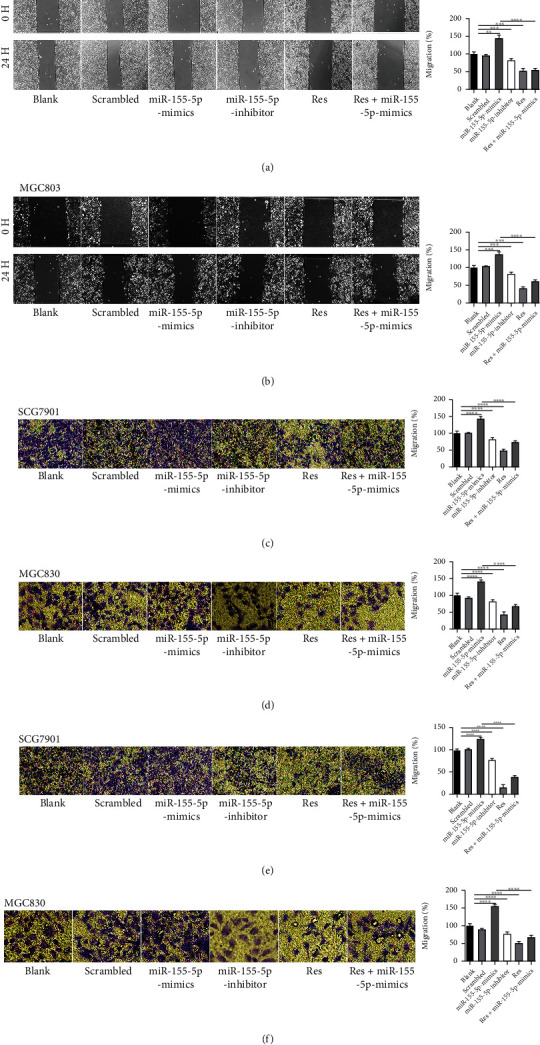
Res inhibits migration and invasion of gastric cancer cells. (a and b) Migration of SGC7901 and MGC803 cells treated with miR-155-5p and 80 *μ*M Res. (c and d) Migration of SGC7901 and MGC803 cells treated with miR-155-5p and 80 *μ*M Res. (e and f) Invasion of SGC7901 and MGC803 cells treated with miR-155-5p and 80 *μ*M Res. ^∗∗^*P* < 0.01, ^∗∗∗^*P* < 0.001, and ^∗∗∗∗^*P* < 0.0001.

Results of wound healing and Transwell assays proved that compared with scrambled controls, miR-155-5p-mimics promoted the motility of gastric cancer cells. However, Res impaired the motility of gastric cancer cells (Figures 3(a)–3(f)).

### 3.4. Res Affects Cell Morphology

**Figure 4:**
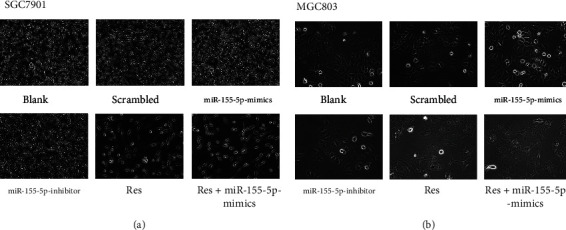
Res change gastric cell morphology (a and b).

The number of adherent cells was significantly increased in the miR-155-5p-mimics group but decreased in the miR-155-5p-inhibitor group; however, neither treatment caused morphological changes in cells. After treatment with Res, the number of adherent cells overexpressing miR-155-5p was significantly reduced, and cells underwent significant changes in morphology (Figures 4(a)–4(b)).

### 3.5. Res Affects the Expression of miR-155-5p Target Genes

**Figure 5:**
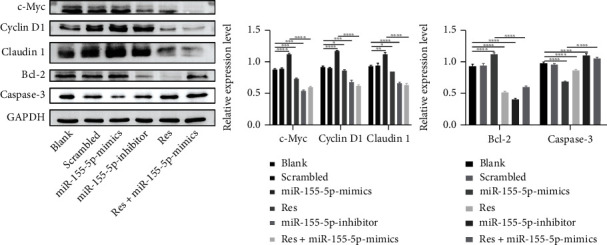
Res regulates the expression of some miR-155-5p-targeted proteins in SGC7901 cells. Western blot analysis was performed to detect the expression of the downstream target protein regulated by miR-155-5p. Data present mean ± standard deviation. ^∗^*P* < 0.05, ^∗∗^*P* < 0.01, ^∗∗∗^*P* < 0.001, and ^∗∗∗∗^*P* < 0.0001.

We selected SGC7901 cell overexpressing miR-155-5p for further experiments. After examining the related scientific literature, three candidate target genes were selected: claudin 1, caspase-3, and c-Myc. We assessed the expression of these genes after treatment with Res. We also examined the expression of proteins regulating cell cycle such as cyclin D1 and the apoptosis-related protein Bcl-2. Western blot analysis showed that miR-155-5p-mimics upregulated claudin 1, c-Myc, cyclin D1, and Bcl-2 expression but downregulated caspase-3 expression. In contrast, Res significantly downregulated claudin 1, c-Myc, cyclin D1, and Bcl-2 expression and upregulated caspase-3 expression, even in the presence of miR-155-5p-mimics (Figure 5).

## 4. Discussion

Gastric cancer is an extremely complex malignancy and involves the activation of proto-oncogenes and inactivation of tumor suppressor genes, as well as the abnormal regulation of related signaling pathways [18]. It is important to elucidate the mechanism underlying gastric cancer progression and identify novel drugs to develop more effective therapeutic approaches. Res is a multitarget antineoplastic drug that exerts strong antitumor effects in different tumor types [19]. For example, in hepatocellular carcinoma, Res inhibits the cell growth via downregulation of MARCH1 expression, and in pancreatic cancer, Res reduces the ability of tumor cells to invade and migrate by inhibiting the expression of miR-21 [20, 21]. However, whether Res can directly suppress miR-155-5p expression in human gastric cancer remained unknown. In this study, we identified that Res inhibited miR-155-5p expression and further influenced claudin 1, cyclin D1, c-Myc, Bcl-2, and caspase-3 expression, thereby blocking the progression of the cell cycle.

miRNAs participate in several processes related to tumor cell invasion and metastasis and are potential noninvasive biomarkers [8, 22, 23]. First, we aimed to identify how Res regulates miR-155-5p expression levels in gastric cancer. We assessed the expression of miR-155-5p in gastric cancer tissues and adjacent tissues.

Accelerated cell cycle progression is a common feature of most solid tumors. To prove that miR-155-5p overexpression can accelerate the cell cycle, we examined changes in the cell cycle of gastric cancer cells after overexpressing or inhibiting miR-155-5p expression in these cells. Our results confirmed that overexpression of miR-155-5p promoted cell proliferation, migration, and invasion and inhibited apoptosis. Thus, miR-155-5p may function as a tumor promoter in gastric cancer cells, consistent with its role in breast, lymphoma, and liver cancers [16, 24, 25].

Subsequently, we used miRNA bioinformatic prediction databases, including miRBase and TargetScan, to further elucidate the molecular mechanism underlying the cellular and biological behaviors of miR-155-5p. Based on our results and the related scientific literature, we selected genes encoding claudin 1, cyclin D1, c-Myc, Bcl-2, and caspase-3 as miR-155-5p-target genes. Claudin 1 is an important component of tight junctions and is abnormally expressed in many different tumors [26–30]. In hepatocellular carcinoma, the deletion of keratin 8 and keratin 18 promotes the proliferation, invasion, and metastasis of HepG2 cells by upregulating claudin 1 expression [31]. Cyclin D1 plays a vital role in cancer pathogenesis as its upregulated expression drives unchecked cellular proliferation [32]. c-Myc acts as a transcription factor and plays a vital role in controlling cell growth, vitality, apoptosis, and cellular transformation [33, 34]. In our results, compared with the control group, the expression levels of claudin 1, cyclin D1, and c-Myc were upregulated in the miR-155-5p-mimics group suggesting that miR-155-5p plays an important role in cell cycle initiation and progression, leading to accelerated proliferation.

Apoptosis is a steady-state process that balances cell survival and death. Liu et al. [34] found that when c-Myc expression was suppressed, cell growth was arrested, and cells accumulated in the G0/G1 phase of the cell cycle, leading to accelerated apoptosis. Activation of caspase-3 is a critical process in apoptosis and increased caspase-3 activity is considered a marker of apoptosis [35]. Bcl-2 is involved in the apoptotic pathway and plays a prominent role in controlling apoptosis and enhancing cell survival [36]. Antineoplastic drugs can inhibit Bcl-2 expression to promote apoptosis [37]. However, targeting only one factor may have limited efficacy in treating malignant tumors [21, 38]. Thus, there is an urgent need to identify drugs that can regulate multiple genes and signal transduction pathways. Researchers have shown that c-Myc, caspase-3, and Bcl-2 play key roles in maintaining cell survival and death. Our results showed that miR-155-5p overexpression upregulated c-Myc and Bcl-2 protein expression and downregulated caspase-3 protein expression. miR-155-5p may inhibit apoptosis by regulating the expression of c-Myc, Bcl-2, and caspase-3.

However, our study had certain limitations. Our results are only based on cell behavior, the detailed molecular mechanisms and cross-talk among claudin 1, cyclin D1, c-Myc, Bcl-2, and caspase-3 warrant further studies. Moreover, our findings need to be verified in animal models to assess whether these results can be extrapolated in humans. In the future, we intend to conduct further research studies to elucidate the mechanism related to the Res-mediated regulation of miR-155-5p expression and its downstream signaling pathways and possibly identify therapeutic markers for gastric cancer.

## 5. Conclusions

We identified that miR-155-5p was overexpressed in gastric cancer and may be a potential molecular target for Res to exert antigastric tumor effects. Our results provide new directions and a theoretical basis for the treatment of gastric cancer in the future.

## Data Availability

The data used to support the findings of this study are available from the corresponding author upon request.
